# Supplementary material for: Resolving the full spectrum of human genome variation using Linked-Reads
Source: Genome Res. 2019 Apr;29(4):635–45. doi: 10.1101/gr.234443.118 (PMC6442396; doi:10.1101/gr.234443.118)
Supplement: Supplemental Material [file supp_gr.234443.118_Supplemental_longranger-2.2.zip › longranger-2.2.tar.bz2!/lr2.2-public/lib/go/src/code.google.com/vitess/web/vtctld2/src/app/status/heatmap.component.html]

## {{keyspace.Name}}

|  |  |  |
| --- | --- | --- |
|  |  |  |

|  |
| --- |
| {{label.CellLabel.Name}} |
|

|  |  |
| --- | --- |
| {{label.CellLabel.Name}} | {{typeLabel.Name}} |
|

Dismiss
